# Improving the estimation of the burden of risk factors: an illustrative comparison of methods to measure smoking-attributable mortality

**DOI:** 10.1186/s12963-015-0039-z

**Published:** 2015-02-19

**Authors:** Peter Tanuseputro, Richard Perez, Laura Rosella, Kumanan Wilson, Carol Bennett, Meltem Tuna, Deirdre Hennessy, Heather Manson, Douglas Manuel

**Affiliations:** Bruyère Research Institute, Bruyère Centre of Learning, Research and Innovation in Long-Term Care, Ottawa, Ontario Canada; Ottawa Hospital Research Institute, Clinical Epidemiology Program, Ottawa, Ontario Canada; Institute for Clinical Evaluative Sciences, Population Health and Primary Care, Ottawa, Ontario Canada; Public Health Ontario, Toronto, Ontario Canada; Dalla Lana School of Public Health, University of Toronto, Toronto, Ontario Canada; Department of Medicine, University of Ottawa, Ottawa, Ontario Canada; Health Analysis Division, Statistics Canada, Ottawa, Ontario Canada; Department of Family Medicine, University of Ottawa, Ottawa, Ontario Canada

**Keywords:** Risk assessment, Risk factors, Population surveillance, Data collection, Burden of illness, Mortality determinants

## Abstract

**Background:**

Prevention efforts are informed by the numbers of deaths or cases of disease caused by specific risk factors, but these are challenging to estimate in a population. Fortunately, an increasing number of jurisdictions have increasingly rich individual-level, population-based data linking exposures and outcomes. These linkages enable multivariable approaches to risk assessment. We demonstrate how this approach can estimate the population burden of risk factors and illustrate its advantages over often-used population-attributable fraction methods.

**Methods:**

We obtained risk factor information for 78,597 individuals from a series of population-based health surveys. Each respondent was linked to death registry (568,997 person-years of follow-up, 6,399 deaths).Two methods were used to obtain population-attributable fractions. First, the mortality rate difference between the entire population and the population of non-smokers was divided by the total mortality rate. Second, often-used attributable fraction formulas were used to combine summary measures of smoking prevalence with relative risks of death for select diseases. The respective fractions were then multiplied to summary measures of mortality to obtain smoking-attributable mortality. Alternatively, for our multivariable approach, we created algorithms for risk of death, predicted by health behaviors and various covariates (age, sex, socioeconomic position, etc.). The burden of smoking was determined by comparing the predicted mortality of the current population with that of a counterfactual population where smoking is eliminated.

**Results:**

Our multivariable algorithms accurately predicted an individual’s risk of death based on their health behaviors and other variables in the models. These algorithms estimated that 23.7% of all deaths can be attributed to smoking in Ontario. This is higher than the 20.0% estimated using population-attributable risk methods that considered only select diseases and lower than the 35.4% estimated from population-attributable risk methods that examine the excess burden of all deaths due to smoking.

**Conclusions:**

The multivariable algorithms presented have several advantages, including: controlling for confounders, accounting for complexities in the relationship between multiple exposures and covariates, using consistent definitions of exposure, and using specific measures of risk derived internally from the study population. We propose the wider use of multivariable risk assessment approach as an alternative to population-attributable fraction methods.

## Background

### Tobacco smoking kills 5.7 million in 2010

The scientific and general media regularly report the number of deaths or cases of disease caused by risks such as smoking, obesity, or physical inactivity. Many clinicians and policymakers use these figures to prioritize and advocate for change at both the individual and population levels. Only a few, however, pause to understand how these estimates are derived, and fewer still consider their limitations.

For example, when the Institute for Health Metrics and Evaluation, following the World Health Organization’s Global Burden of Disease reports, estimated 5.7 million deaths in 2010 were caused by tobacco smoking [1], many may assume these deaths were directly observed in smokers. Many may also assume that researchers have sufficiently teased out the independent effects of interrelated risk factors, such as physical inactivity and obesity. In fact, while an abundance of work has been done to produce valid estimates, there are limitations to commonly used methods to estimate the population burden of risk factors.

### How is the population burden of risk factors typically estimated?

Population-attributable fraction methods are commonly used to estimate the burden of risk factors. Typically, these methods combine summary estimates of the following: prevalence of risk factor exposure, hazards relating exposure and burden outcome, and counts of outcome (see methods for formulas). The estimate of 5.7 million deaths caused by tobacco smoking was not generated by comparing worldwide counts of deaths in smokers and non-smokers, but rather by combining prevalence estimates of smoking with smoking hazards from the literature and then multiplying the proportions with mortality counts for each country.

### Do we need an alternate method?

The calculation of population-attributable fractions is useful in jurisdictions with limited population-level data since hazards (relative risks) can be obtained from the literature and prevalence of exposure, if not available, can be inferred from similar populations or from known associations between certain exposures and outcomes (i.e. “indirect” methods) [1-3]. However, population-attributable fraction methods also have many limitations [2-4].

Many of the issues and limitations of population-attributable fraction methods stem from using and combining *ecological*, summary measures of exposure, outcome, and hazard, across different sources of data:

• *Exposure mismatch:* Definition of exposure categories (e.g., current and former smoking) across data-sources for prevalence and hazard often differ.
• *Confounding:* Often uses adjusted hazards from the literature. This is limited by the availability of adequate studies or reviews.
• *External generalizability:* Choosing hazards is complicated by heterogeneity among available studies. Studies use differing sets of adjusted variables, varying exposure definitions and distributions, and populations with varying baseline risks, which may not be similar to the target population.
• *Imprecision:* Even when well-adjusted hazards are available (e.g., through meta-analytical techniques), the application of external summary hazards to the population of interest is imprecise. The distribution of potential confounders in the population of interest is ignored as individual-level adjustment is not possible.

### New opportunities with linked exposure and outcomes data

The single most important data requirement for improving estimates of risk factor burden in a population are data systems that include population-based cohorts (with information on various risk factor exposures) who are followed for health outcomes. When such data are available, it becomes possible to directly estimate the incidence of disease in people both exposed and not exposed to risk factors, opening up opportunities to measure attributable burden without relying on population-attributable fraction methods.

In health care systems with a dominant payer (i.e., government or a conglomerate of funders), administrative databases routinely collect information on health and service use at a population level. As research infrastructure and information technology develop, jurisdictions are increasingly able to link individual-level data captured in these databases, creating combined datasets with a longitudinal array of information on health care use and outcomes. Similarly, detailed information on risk factor exposures is increasingly collected in cross-sectional surveys representative of the population. Examples include the United States National Health Interview Survey, the Health Survey for England, the Australian Health Survey, and various health surveys in Scandinavian countries. In Ontario, Canada, the Canadian Community Health Surveys are linked at an individual level to health care use and outcomes databases. Other provinces and countries are developing similar linked data repositories.

### Multivariable models

Individual-level population-based data linking exposures to outcomes can be used to generate multivariable algorithms. These models estimate an individual’s probability of an outcome given a set of exposures. Exposure-outcome combinations are modeled when there is a well-established causal relationship, such as between smoking and death. Potential confounding variables can be included and interaction terms can be explored. The appropriate statistical model (e.g., logistic regression, linear regression, Cox proportional hazards) will be dependent on the nature of the outcome variable (e.g., dichotomous, continuous, time to event) and its relationship to the independent exposure variables.

The use of such multivariable methods to estimate risk factor burden is still rare [5,6]. The main benefit of this approach is that it uses an internal, cohesive, individual-level data source for the inputs of exposure, risk, and burden.

### Comparing methods for burden estimation

Our manuscript uniquely describes the use of predictive, multivariable algorithms for the purpose of measuring population-attributable burden and is the first to directly compare such methods to population-attributable fraction techniques. Using smoking-attributable mortality as a case example, we present estimates derived from a single set of population data sources (using a multivariable risk assessment approach) and compare them with those generated by traditional population-attributable fraction techniques.

## Methods

We used three methods for estimating smoking-attributable mortality. First, we created multivariable predictive algorithms for death and applied them to the risk profile of the population. We compared these estimates with those calculated using the original formulas for attributable-fraction, and also from widely used population-attributable fraction techniques that combine summary measures of prevalence, relative risk, and mortality.

### Data sources

We obtained prevalence estimates of smoking and other behavioral risk factors from the Ontario sample of the Canadian Community Health Survey (CCHS). The CCHS provides ongoing cross-sectional estimates of health determinants, health status, and health system utilization at a sub-provincial level in two-year cycles. The target population of the CCHS includes household residents over 12 years of age in all provinces and territories, with the principal exclusion of populations on Indian reserves, Canadian military bases, and some remote areas. Respondents to the CCHS were linked to the Registered Persons Database (RPDB), containing information on births and deaths in Ontario.

The Cancer Prevention Study (CPS) II is a prospective study of 1,185,106 adults (at baseline) in the United States over the age of 30 years [7]. Disease-specific relative risk estimates for current and former smokers as compared to never smokers were obtained from CPS II, which have often been used to estimate other estimates of smoking-attributable mortality using population-attributable fraction methods. We used updated relative risks mainly derived from CPS II, published in the 2014 Surgeon General’s report (Table 12.3), that have increased the number of age-groupings and considered more recent datasets [8].

### Multivariable algorithms for mortality

**Figure 1:**
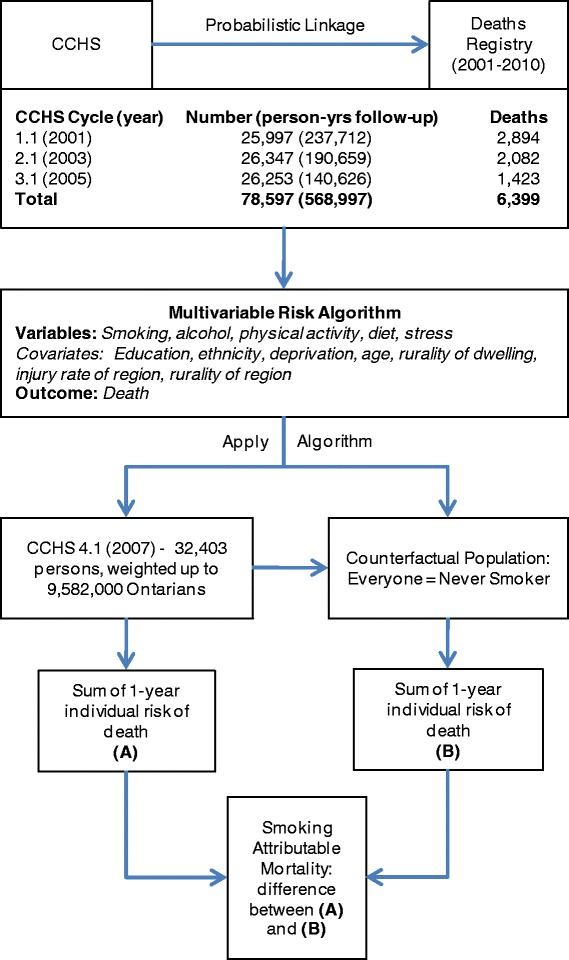
**Generation of multivariable risk algorithm for death based on smoking, other health behaviours, and covariates from the Canadian Community Health Survey (CCHS).**

We examined three cycles of the CCHS (2001, 2003, 2005) and using linkage to RPDB observed for any deaths from the time of entry into the CCHS (Figure 1). Individuals were excluded if they did not agree to have their records shared with the provincial government, if they could not be linked to the death registry (using a probabilistic algorithm), if their health card became ineligible (signifying possible migration), or if the records had missing values for variables of interest.

The linked dataset was used to generate sex-specific, age-adjusted Cox proportional hazard models for death. This type of regression was chosen because the primary outcome was time to death and due to the need to account for censoring. Smoking was included as one of the main predictor variables in the model, with the exposure levels of daily current heavy smoker (≥1 pack/day), daily current light smoker (<1 pack/day), former smoker, and never smoker. These levels of exposure were selected to allow comparisons to attributable-fraction methods and to other studies. Other health behavior predictor variables in this algorithm were: leisure physical inactivity (total metabolic equivalent of task (MET)/day for a list of activities), diet (index based on consumption of fruit and vegetables, potatoes, fruit juice, and carrots), alcohol use (drinks/week and bingeing), and stress (self-rated single question). We also included in the model variables related to age (time-varying covariate, including a spline to account for rapid non-linear increase in mortality in older ages), ethnicity (Caucasian vs. non-Caucasian), education, rurality, neighborhood deprivation [9,10], and regional injury hospitalization rates as they improved the discrimination and/or calibration of the model.

The risk algorithms were applied to the risk profile of the 2009 CCHS respondents to predict the 1 year mortality of the current population. We then applied the algorithm to a counterfactual population where no-one smoked (i.e. all current and former smokers were re-classified as a never smoker). The absolute difference between the total deaths in the actual and counterfactual population is the smoking-attributable mortality (Figure 1).

We verified the predictive ability of our algorithm by calculating the c-statistic, a measure of discrimination of the model (i.e., its ability to resolve the population into those that will die versus those that will not die). We assessed accuracy of the model by comparing observed and predicted risk across deciles of risk and calculated the Hosmer-Lemeshow chi-square. We also validated our model by examining how well it predicts death across subgroups of variables not included in the model (e.g., income and body mass index). More details of our method can be found elsewhere [11].

### Direct observation of excess deaths due to smoking– Levin’s method

Population-attributable fraction (AF_p_) estimates the proportion of disease that can be attributed to a particular risk factor. The concept of AF_p_ was first expressed by Levin in 1953 as:

$$ {\mathrm{AF}}_{\mathrm{p}}=\left({\mathrm{I}}_{\mathrm{t}}\hbox{--}\ {\mathrm{I}}_{\mathrm{u}}\right)/{\mathrm{I}}_{\mathrm{t}}, $$

where I_t_ is the incidence rate of the outcome in the total target population and I_u_ the outcome rate in those who are unexposed [12]. The use of this formula is referred to as Levin’s method. Multiplying AF_p_ by mortality counts generates population-attributable mortality estimates.

We calculated AF_p_ using Levin’s method by inputting the mortality rate in the Ontario population (I_t_) and the mortality rate for non-smokers (I_u_) as obtained using the linked CCHS data. We calculated AF_p_ for each sex and five-year age groups and multiplied the fractions by the number of all-cause deaths in Ontario within each category. The age-/sex-specific smoking-attributable mortality counts are then summed to obtain the total smoking-attributable mortality.

### Population-attributable fraction method

Burden estimates are not typically calculated using Levin’s original method because the necessary data may not exist. For many populations, we do not know the incidence of disease broken down by individual risk exposure. Whether a person smokes, drinks, or is obese is not typically recorded in death or disease registries. Risk factor exposure is infrequently collected as part of population-level outcomes data. Consequently, current population-based estimates of disease incidence in the exposed (I_e_) versus unexposed (I_u_), are often not available.

Instead, AF_p_ is usually derived mathematically by combining the prevalence of the risk factor (P_e_) in the target population with a measure of association between the risk factor and burden (relative risk, RR), obtained from epidemiological studies. Past and recent efforts (e.g., Global Burden of Disease reports [1], Centers for Disease Control and Surgeon General smoking-attributable mortality estimates [7,8]) to estimate the population burden of disease risk factors have used this technique, modifying the basic formula for a dichotomous exposure [4,8,13-16]:

$$ {\mathrm{AF}}_{\mathrm{p}} = \left[{\mathrm{P}}_{\mathrm{e}}\left(\mathrm{R}\mathrm{R}-1\right)\right]/\left[1+{\mathrm{P}}_{\mathrm{e}}\left(\mathrm{R}\mathrm{R}-1\right)\right] $$

We estimated AF_p_ for smoking by combining prevalence estimates from the CCHS and disease-specific relative risks for smoking from the Cancer Prevention Study II (CPS-II), published in the 2014 Surgeon General report [8]. We did so for each disease thought to be causally linked to smoking [8], using AF_p_ formula modified for multiple exposure groups:

$$ {\mathrm{AF}}_{\mathrm{p}} = \left[\left({\mathrm{p}}_0+{\mathrm{p}}_1{\mathrm{xRR}}_1+{\mathrm{p}}_2{\mathrm{xRR}}_2\right)-1\right]\ /\left[{\mathrm{p}}_0+{\mathrm{p}}_1{\mathrm{xRR}}_1+{\mathrm{p}}_2{\mathrm{xRR}}_2\right] $$

where p_0_, p_1_, and p_2_ represent the percentage prevalence of never, current, and former smoking, while RR_1_ and RR_2_ represent the relative risk of death due to a given disease for current and former smokers, respectively, with never smokers serving as the reference group and counterfactual population. The AF_p_ for each disease is multiplied by the number of deaths in the population from each respective disease to obtain cause-specific smoking-attributable mortality estimates.

For each disease group, smoking-attributable mortality estimates were estimated for males and females and by the four age groups (35–54, 55–64, 65–74, and 75+ years) used in the CPS II relative risk estimates [8] and summed to generate the total smoking-attributable mortality.

## Results

### Multivariable algorithms

Out of the 123,821 Ontario respondents from the three cycles of CCHS, 78,597 individuals were linked to the deaths registry (RPDB) and had adequate information for use in the algorithm (Figure 1). A total of 568,997 person-years of follow-up were observed, including 6,399 deaths. The hazards for smoking for heavy current smokers, light current smokers, and former smokers were 2.8 (95% CI: 2.4, 3.2), 2.2 (95% CI: 1.9, 2.5), and 1.4 (95% CI: 1.3, 1.5) for males and 2.9 (95% CI: 2.6, 3.4), 2.2 (95% CI: 2.0, 2.5), and 1.7 (95% CI: 1.5, 1.8) for females, respectively (Table 1). The hazards for other parameters in the model can be found elsewhere [11].

**Table 1:** **Characteristics of study population - CCHS 4.1 (2009), Ontario, age 20 years+**

**Sex**	**Smoking status**	**Population count (n)**	**Prevalence (%)**	All-cause mortality hazard ratios*
Males	Heavy current smoker	389 364	8.4	2.8 (2.4, 3.2)
	Light current smoker	802 080	17.3	2.2 (1.9, 2.5)
	Former smokers	1 273 064	27.5	1.4 (1.3, 1.5)
	Never smokers	2 168 608	46.8	Reference
	Total	4 633 116	100	
Females	Heavy current smoker	182 738	3.8	2.9 (2.6, 3.4)
	Light current smoker	701 209	14.4	2.2 (2.0, 2.5)
	Former smokers	974 084	20.0	1.7 (1.5, 1.8)
	Never smokers	3 012 968	61.9	Reference
	Total	4 870 999	100	

*Hazard ratios calculated from model derived using Canadian Community Health Survey (CCHS) cycles 1.1-3.1, adjusting for physical inactivity, unhealthy eating, stress, alcohol, and other covariates. Hazard ratios are followed by 95% confidence intervals in brackets.

The CCHS 4.1 population had a combined total of 9,582,000 weighted individuals (Table 1). The age-adjusted Cox proportional hazard model for males and females was applied to the CCHS 4.1 population to predict a total of 68,200 deaths in the following year. The counterfactual population where each individual was a never smoker produced 26.1% and 21.4% fewer deaths for men and women, for smoking-attributable mortality estimates of 11,332 and 9,285, respectively (Table 2). This produced a total AF_p_ of 23.7% and smoking-attributable mortality of 20,573.

**Table 2:** **Comparison of three methods for estimating smoking-attributable fraction and mortality**

	**Multivariable predictive algorithm**	**Levin's Method**	**Population-attributable fraction**
*Method comparison*			
**Overview**	Multivariable models relating exposure (and covariates) to outcome are created, then applied to current exposure data in the target population to predict total burden. The models may be created from earlier years of data from the same target population.	Rate of outcome in the total population is compared to the rate in the unexposed population to estimate the contribution of exposure to excess outcome.	Prevalence of exposure in the target population is combined with hazards relating exposure to outcome from an etiologic study. This is done to estimate proportion of burden attributable to the exposure in the population.
**Computational method**	The models are applied to a counterfactual population where no one is exposed	(AF_p_) = (I_t_-I_u_)/I_t_, AF_p_ is multiplied by total outcome count (see text)	AF_p_ = [P_e_(RR-1)]/[1 + P_e_(RR-1)], AF_p_ multiplied by total outcome count (see text)
**Typical data Source**	Population-based, routinely collected data on health outcomes that are linked at the individual level to exposure data, often from health surveys.	Not commonly available at the population level. Cohort studies, disease registries, or exposure data linked to outcome.	Ecological, summary measures of: prevalence from health surveys, hazards from the literature, and outcome counts from routinely collected data.
*Study data sources*			
**Smoking prevalence (target population)**	Canadian Community Health Survey (CCHS) 4.1	Not used	CCHS 4.1
**Hazard estimates**	CCHS 1.1 to 3.1 linked to death database	Not used	Cancer Prevention Study II, 2014 Surgeon General’s Report [8]
**Mortality estimates**	Predicted by algorithm	CCHS 1.1 to 3.1 linked to death database	Death database (RPDB)
*Smoking-attributable fraction/deaths, 2009–2010*			
***Males***			
**Smoking-attributable fraction (AF**_**p**_ **)**	26.1%	36.8%	24.1%
**Smoking-attributable mortality (SAM)**	11 332	15 998	10 648
***Females***			
**AF**_**p**_	21.4%	33.9%	15.8%
**SAM**	9 285	14 713	6 928
***Total***			
**AF**_**p**_	23.7%	35.4%	20.0%
**SAM**	20 573	30 711	17 576

The models satisfied Cox proportional hazards assumptions and produced a c-statistic of 0.87 for both sexes. Calibration of the model was excellent across deciles of risk of body mass index categories, income levels, and 14 Local Health Integration Networks responsible for health planning and resource allocation in Ontario (data not shown).

### Attributable-fraction techniques

Table 2 highlights differences between the three methods used. When compared to estimates from the predictive algorithm, direct observation of the excess mortality in smokers (Levin’s formula) led to a larger total smoking-attributable mortality of 30,711 and AF_p_ of 35.4% (Table 2). Males again produced greater attributable mortality and AF_p_ than females. Conversely, conventional AF_p_ techniques combining prevalence from the CCHS and relative risks for specific causes of death from CPS II yielded a smaller total AF_p_ of 20.0% and smoking-attributable mortality of 17,576.

## Discussion

This study has shown that burden estimates can vary considerably depending on the method used. Different methods are feasible for different levels of data availability. We have presented a multivariable modeling method commonly used in clinical settings (e.g., Framingham risk scores for heart disease), that is enabled when individual-level data are collected at a population level from exposure to outcome.

### Strengths of the multivariable risk approach

The multivariable risk approach has several advantages. *First,* there are difficulties with the interpretation of AF_p_ across risk factors in population-attributable fraction methods. As discussed, hazards (RRs) used in the calculation of AF_p_ are often inadequately or inconsistently adjusted for confounding, interaction, or mediation; thus, AF_p_ for multiple risk factors, estimated individually, can add up to more than 1 [4,17,18]. On the contrary, the multivariable modeling method allows simultaneous inclusion of multiple risks, along with relevant confounding variables and interaction terms at the level of the individual. In our case example, we concurrently examined the burden of physical inactivity, unhealthy diet, alcohol, and stress while controlling for numerous confounders (Figure 1).

Advanced attributable fraction methods that calculate *partial* population-attributable risk typically allow similar exploration in case–control and cohort studies [19-23]. These methods can potentially be modified for use in population-based studies of burden, but this again necessitates linkage of population-based exposure and outcome data at the individual level.

In our case example, Levin’s original formula led to higher estimates of smoking-attributable mortality compared to those from the predictive algorithm. This is likely because Levin’s method simply examines the difference between all-cause mortality in smokers and non-smokers and does not account for the potential confounders (e.g., alcohol use or socioeconomic position) included in the predictive algorithm, thus inflating smoking’s observed effect on death.

In contrast, estimates derived from the multivariable algorithms were higher than those from population-attributable fraction methods, which notably used disease-specific estimates of relative risk. Excluded are deaths from diseases for which robust etiologic studies are not available. The use of disease-specific hazards is particularly important in population-attributable risk methods since potential confounders are typically not fully accounted for. This increases the need for caution when including diseases for which smoking may have a smaller independent contribution. Conversely, in using robust population-based data to generate a multivariable algorithm with high levels of discrimination, we can have greater comfort in including deaths from all causes to estimate attributable burden.

*Second,* linking exposure data directly to outcomes allows the use of hazards that have been observed *within* the target population. This avoids the issues of external generalizability faced when hazards from etiologic studies are used in population-attribution fraction methods.

*Third,* the use of linked data allows consistent definitions of exposure in measures of exposure prevalence and disease hazards. For smoking, the level of consumption before someone is deemed a current or former smoker varies considerably in both health and etiologic surveys. Such mismatch can lead to considerable error in burden estimation [3]. Multivariable models allow meaningful exposure categorization that reflects the distribution of exposure and hazard in the target population, rather than trying to match the definitions used in the hazard studies. For smoking, risk in the population will be dependent not only on how one classifies a current or former smoker (typical categories in etiologic studies) but also on factors such as smoking intensity, duration, and time since quitting.

*Fourth,* using individual-level data avoids the imprecision of combining summary measures of exposure, risk, and outcome across data sources [9,10]. For example, the inputs for traditional methods are often not powered to produce precise age- and sex-specific AF_p_. For smoking, where both exposure and outcome vary significantly by age and sex, using summary measures can lead to biased estimates [3].

In most developed countries where the prevalence of smoking decreases with increasing age, using broad age-groups for AF_p_s will lead to an overestimation at older ages. Overall, smoking-attributable mortality is in turn overestimated since mortality increases with age. The 2014 Surgeon General Report’s relative risk estimates [8] has attempted to address this with the introduction of four age-groups (compared with two for select diseases in the 1989 report [7]). The new set of relative risk estimates, used for the population-attributable fraction method in this study, also considered more recent follow-up data and included new disease groupings. When compared to estimates generated using relative risks published in the 1989 report (1982–1988 follow-up), overall AF_p_ increased from 18.5% to 20.0%, with a resulting increase of smoking-attributable mortality from 15,803 to 17,576.

*Finally,* estimating disease incidence in the exposed population enables the use of other measures that are potentially more meaningful, such as reduced life expectancy and health-adjusted life expectancy, for those exposed and for the entire population. In addition, there are further benefits in the application of multivariable models to time periods and regions beyond the original derivation population:

• Validated and calibrated multivariable models can be applied to current exposures to approximate future burden. Past exposure levels can be applied to estimate current burden, accounting for the varying lag time between exposures and outcomes [3].
• Considerable advances in methods, including in the calibration of predictive models, allow multivariable algorithms to be applied to a range of populations beyond the original study population.
• Case example: the prediction model created in Ontario could be applied to other Canadian provinces that participated in the same health survey.
• This opens the door for jurisdictions without linked data to estimate disease incidence based on exposure to risk factors.
• It is becoming increasingly apparent that it is possible to use routinely collected population data to accurately assess baseline risk (or disease incidence) that is well-calibrated for risk factor exposures, as measured in population health surveys.

Over the past 30 years, predictive risk algorithms have been developed for application purposes in the clinical setting, albeit using clinical data and with a focus on risk stratification.

### Limitations of the multivariable risk approach

Multivariable approaches to building prediction models and estimating attributable fraction are well-established methods. Our main contribution is their application to estimate risk factor burden in the population.

One limitation of our approach is that our estimates are conditional on the assumptions and validity of our predictive models, which could change over time, necessitating revision and recalibration. Our model, along with the population-attributable fraction method, uses *baseline* estimates of exposures, as necessitated by the cross-sectional nature of the population-level data. Thus, we cannot take into account time-varying exposures and confounders [21-23].

Recent advances in attributable fraction methods are also based on multivariate techniques and take advantage of longitudinal studies, where repeated exposure and outcome data are available at the individual level. These advances include marginal structural models and g-estimation, to account for time-varying covariates, and longitudinal extensions of the average attributable fraction method [20-22]. Such longitudinal studies, however, rarely occur at a population level and are thus unable to provide population burden estimates.

As with all risk-attribution methods, we were also limited in our ability to infer causality by unmeasured confounders. Nevertheless, we should continually attempt to create models that best represent known causal relationships and to build in complexities as more information is obtained. More advanced modeling techniques, such as hierarchical modeling and multilevel growth models [24], can be increasingly incorporated as our understanding of causal pathways improves and more detailed data are available at a population level.

Predictive models must be built and interpreted cautiously when they are intended to infer causal relationships [17,25]. Included exposures and covariates should be strongly suspected of having causal relationships to the outcome. Overly complex models, with variables added solely to improve predictive precision, should be avoided to prevent overfitting, where the model describes random error. Predictive performance, poor in overfitted models, should be explored. Furthermore, causal pathways should be considered, and variables directly along the causal pathway should generally be excluded to avoid reducing the hazard of the studied exposure. The model-building approach for our case example focuses on distal behavioral variables. We did not sacrifice theoretical accuracy for improved prediction.

## Conclusions

Significant resources are invested globally in the collection of data on risk factors. Many data systems, however, continue to focus on producing prevalence estimates in the population, without planned linkage to health outcomes. This necessitates the use of population-attributable fraction methods. We present a method that circumvents their well-documented limitations [2-4].

**Figure 2:**
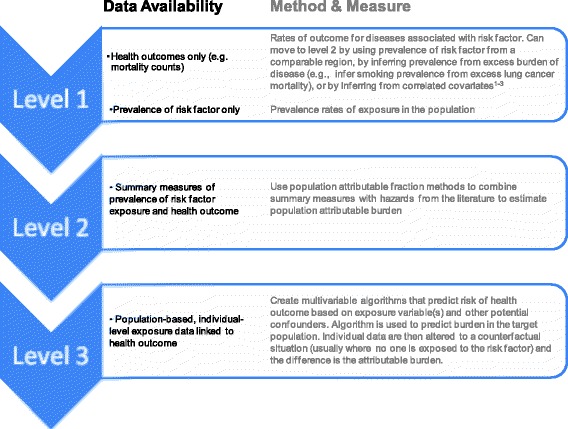
**Methods to estimate the burden attributed to risk factors, based on the levels of data availability.**

We recognize that many regions do not have the data and resources required to produce population-based predictive algorithms. Consequently, the method used to estimate the burden of risk factors will be dependent on the level of data development (Figure 2). Nevertheless, systems for collecting data on risk factors should strive to create population-based databases linking exposure and outcome at the individual level. Linkages to health administrative databases will enable modeling of outcomes beyond mortality, such as progression to disease, hospitalizations, and health care costs. Linkages with non-health databases will allow estimation of disease burden related to the broader determinants of health.

Global and regional burden of disease projects should consider using the best available method for each population. Gross differences in estimates between methods can be reported as sensitivity analyses if comparisons across jurisdictions with differing data availability are performed. Individual regions such as Canada (CCHS), the United States (National Health Interview Survey) [5,6], England (Health Survey for England), and India (the Million Deaths study) [26] that capture linked exposure and outcome data at the individual level should attempt to build multivariable models to estimate burden. In addition, further work may explore the correct application of multivariable algorithms to external populations, following validation and calibration.

Clinical medicine uses multivariable risk algorithms, such as the Framingham risk score, to predict outcomes for individuals and show the combined contribution of risk factors. Population health tools measuring population burden from risk factors should strive to do the same.

## Abbreviations

CCHS: Canadian community health survey
CPS: Cancer prevention study
AF_p_: Population-attributable fraction
RPDB: Registered persons database
RR: Relative risk
